# Data on potential of CO_2_ capture and enhanced water recovery projects in modern coal chemical industries in China

**DOI:** 10.1016/j.dib.2019.103810

**Published:** 2019-03-02

**Authors:** Ning Wei, Xiaochun Li, Zunsheng Jiao, Shengnan Liu, Robert Dahowski

**Affiliations:** a State Key Laboratory for Geo-mechanics and Geo-technical Engineering, Institute of Rock and Soil Mechanics, Chinese Academy of Sciences, Wuhan, 430071, Hubei Province, China; b School of Energy Resources, University of Wyoming, Laramie, 82071, Wyoming, USA; c Pacific Northwest National Laboratory, Richland, WA, USA

## Abstract

This data article presents the first systematic assessment on the potential of onshore CO_2_-enhanced water recovery (CO_2_-EWR) using pure CO_2_ streams from industrial separation processes by this evaluation framework. The evaluation framework is developed for CO_2_ capture, geological utilization and storage (CCUS) project developments, including CO_2_ emission inventory, site suitability evaluation, and source–sink matching with techno-economic models. The data shows the matched source (CO_2_ source)-sink (onshore aquifer site) pairs with sites distribution and levelized cost under various scenarios. Data also shows the geographic distribution of source-sink pairs, cost curve, annual cumulative CO_2_ storage capacity and enhanced water production under various scenarios. Potential large-scale deployments of CO_2_-EWR projects with low cost in the modern coal chemical industry in China are shown and identified in the dataset. This data article is related to the research article “Cost curve of large-scale deployment of CO_2_-enhanced water recovery technology in modern coal chemical industries in China” (X. Li et al. 2019).

**Table:** Specifications table

Subject area	*Chemistry, Energy, Economics*
More specific subject area	*CO*_*2*_*capture, geological utilization and storage*
Type of data	*Tables and figures*
How data was acquired	*An evaluation framework is developed for CCUS project developments, including evaluation of CO*_*2*_*sources, site suitability, and source–sink matching with techno-economic model. The framework was used to evaluate large CO*_*2*_*sources in the modern coal chemical industry and the distribution of suitable onshore aquifer sites in China, and then to obtain the cost curve of potential CCUS projects through source-sink matching analysis. Finally, potential for cost-effective and large-scale deployment of CO*_*2*_*-EWR projects is identified for the coal chemical industry in China.*
Data format	*Systematically analyzed*
Experimental factors	*Numerical simulation*
Experimental features	*Table shows the matched source-sink pairs with spatial distributions and potential costs under various scenarios*
Data source location	*Institute of Rock and Soil Mechanics, Chinese Academy of Sciences* *No 2, Xiaohongshan, Wuhan, Hubei 430071, China*
Data accessibility	The Data is available with this article
Related research article	Li X, Wei N, Jiao Z, Liu S, Dahowski R. Cost curve of large-scale deployment of CO_2_-enhanced water recovery technology in modern coal chemical industries in China. International Journal of Greenhouse Gas Control. 2019; 81:66–82 [1].

**Table:** 

**Value of the data** • This data provides a cost curve for full-chain CCUS projects in the coal chemical industry in China based on a developed systematical evaluation framework, including evaluation of CO_2_ sources, site suitability, and source–sink matching with techno-economic models; • *Large CO*_*2*_ *sources in several processes in the coal chemical industry and matched suitable onshore aquifer sites are inventoried in the data;* • *The cost curve of onshore CO*_*2*_*-EWR in several coal chemical processes in the modern coal chemical industry in China is evaluated and mapped in the datasheet;* • *The data shows various potential large-scale deployment of CO*_*2*_*-EWR projects using pure CO*_*2*_ *from the coal chemical industry in China.*

## 1 Data

**Fig. 1:**
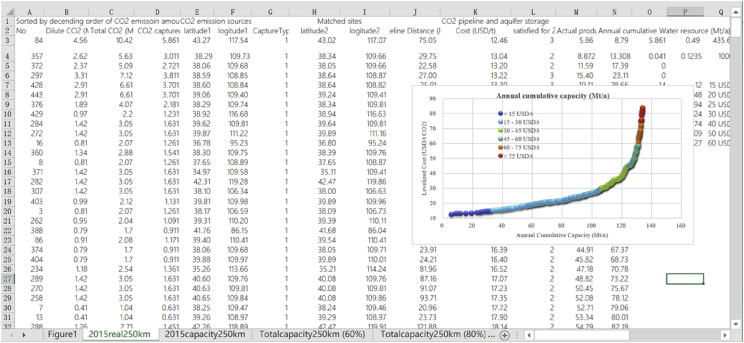
Spreadsheet tabs with figures (excerpt).

**Fig. 2:**
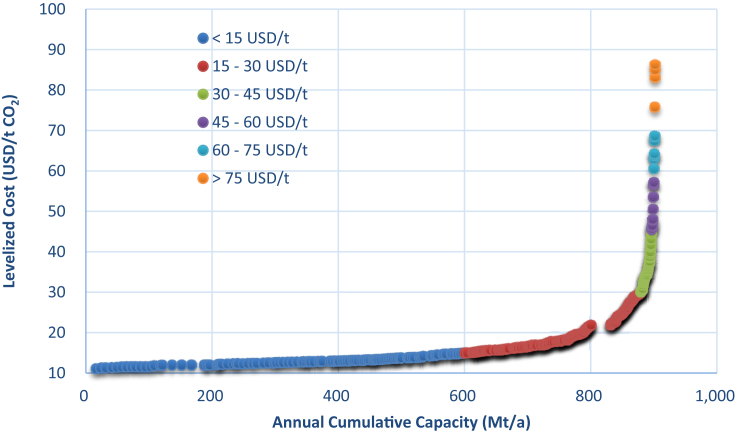
Cost curve of possible full-chain CCUS project versus annual cumulative capacity based on total capacity in 2016 (250-km searching range).

*The data is on the first macro-scale study of potential CO*_*2*_ *capture and CO*_*2*_*-EWR projects by developed source-sink matching method with budget-type techno-economic models for coal chemical industry in China. The data show the details of matched source-sink pairs with potential costs.* The data compiled in excel spreadsheets provides the inventory of several coal chemical processes, CO_2_ emission from industrial and dilute processes of each CO_2_ source, geographic distribution of potential integrated CCUS projects (source-sink pairs) with levelized costs and CO_2_ storage capacity, cumulative storage capacity and water production for the whole set of modeled CO_2_ sources and onshore aquifer sites in China under various scenarios of actual operating capacity in 2015, designed full capacity in 2015, and total capacity in 2016. The data can be used to identify various potential low-cost large-scale deployment of CO_2_-EWR projects using pure CO_2_ from the coal chemical industry in China. Data sheet and figures are compiled using Excel software (Spreadsheet tabs are shown in Fig. 1 and cost curve of one scenario is shown in Fig. 2).

## 2 Experimental design, materials and methods

The systematical evaluation framework is based on the *synthetic framework including evaluation of CO*_*2*_ *sources, site suitability, and source–sink matching with techno-economic models*, and cost curve of potential integrated CCUS projects [2], [3], [4], [5]. The details of this framework can refer to the paper by Li, Wei [1]. The data is generated through this framework considering lots of factors, such as, technology design of full-chain CCUS project, technical features of coal chemical factory, economic parameters, factory location, project size, compression technology, location of storage sites, geological features, injection strategy, reservoir capacity, injectivity of wells, and potential for enhanced resource recovery for each prospective reservoir. The data generated by this framework include the inventory of several coal chemical processes, CO_2_ emission from industrial and dilute processes, spatial distribution of potential integrated CCUS projects (source-sink pairs) with levelized costs, cumulative storage capacity and water production for the whole set of modeled CO_2_ sources and onshore aquifer sites.
